# Hexakis(μ_2_-2-amino­ethanethiol­ato)­triiron(III) tris(perchlorate)

**DOI:** 10.1107/S1600536808036167

**Published:** 2008-11-08

**Authors:** Asako Igashira-Kamiyama, Takumi Konno

**Affiliations:** a Department of Chemistry, Graduate School of Science, Osaka University, Toyonaka, Osaka 560-0043, Japan

## Abstract

In the title salt, [Fe_3_(C_2_H_6_NS)_6_](ClO_4_)_3_, the trinuclear cation lies on a special position of 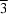 site symmetry; the central Fe atom is coordinated by six thiol­ate groups from the two flanking *fac*-(*S*)-[Fe(C_2_H_6_NS)_3_] units. In the flanking units, the three C_2_H_6_NS groups each chelate to the metal atom. The cations inter­act with the perchlorate anions through weak N—H⋯O hydrogen bonds resulting in a three-dimensional network. In the asymmetric unit two cations are present, one of which is disordered over two positions with occupancies of 0.75 and 0.25.

## Related literature

For related structures, see: Busch & Jicha (1962 ▶); Heeg *et al.* (1985 ▶); Mahboob *et al.* (2004 ▶); Marsh *et al.* (1986 ▶); Matsuura *et al.* (2006 ▶). 
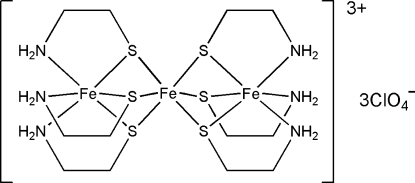


## Experimental

### 

#### Crystal data

[Fe_3_(C_2_H_6_NS)_6_](ClO_4_)_3_
*M* *_r_* = 922.73
Trigonal, 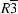

*a* = 14.2852 (6) Å
*c* = 26.2187 (8) Å
*V* = 4633.6 (2) Å^3^
*Z* = 6
Mo *K*α radiation
μ = 2.12 mm^−1^
*T* = 200 (2) K
0.20 × 0.20 × 0.10 mm

#### Data collection

Rigaku R-AXIS RAPID diffractometer
Absorption correction: multi-scan (*ABSCOR*; Higashi, 1995 ▶) *T*_min_ = 0.677, *T*_max_ = 0.816
15327 measured reflections
2365 independent reflections
2144 reflections with *I* > 2σ(*I*)
*R*_int_ = 0.053

#### Refinement

*R*[*F* ^2^ > 2σ(*F* ^2^)] = 0.032
*wR*(*F* ^2^) = 0.100
*S* = 1.39
2365 reflections
165 parameters
6 restraints
H-atom parameters constrained
Δρ_max_ = 1.09 e Å^−3^
Δρ_min_ = −0.31 e Å^−3^

### 

Data collection:*PROCESS-AUTO* (Rigaku, 1998 ▶); cell refinement: *PROCESS-AUTO*; data reduction: *CrystalStructure* (Rigaku/MSC, 2004 ▶); program(s) used to solve structure: *SIR97* (Altomare *et al.*, 1999 ▶); program(s) used to refine structure: *SHELXL97* (Sheldrick, 2008 ▶); molecular graphics: *ORTEP-3 for Windows* (Farrugia, 1997 ▶); software used to prepare material for publication: *Yadokari-XG* (Wakita, 2000 ▶).

## Supplementary Material

Crystal structure: contains datablocks I, global. DOI: 10.1107/S1600536808036167/ng2504sup1.cif

Structure factors: contains datablocks I. DOI: 10.1107/S1600536808036167/ng2504Isup2.hkl

Additional supplementary materials:  crystallographic information; 3D view; checkCIF report

## Figures and Tables

**Table 1:** Selected bond lengths (Å)

Fe1—S1	2.2764 (5)
Fe2—N1	2.0482 (18)
Fe2—S1	2.2434 (6)
Fe3—S2*B*	2.281 (2)
Fe3—S2	2.2869 (7)
Fe4—N2	2.026 (4)
Fe4—N2*B*	2.059 (11)
Fe4—S2*B*	2.229 (2)
Fe4—S2	2.2535 (8)

**Table 2:** Hydrogen-bond geometry (Å, °)

*D*—H⋯*A*	*D*—H	H⋯*A*	*D*⋯*A*	*D*—H⋯*A*
N1—H1⋯O2	0.92	2.28	3.130 (3)	154
N1—H2⋯O3^iii^	0.92	2.40	3.162 (3)	140
N2—H7⋯O1^ii^	0.92	2.30	3.110 (4)	147
N2—H8⋯O2	0.92	2.39	3.274 (4)	161
N2*B*—H13⋯O2	0.92	2.41	2.984 (12)	121
N2*B*—H14⋯O1	0.92	2.27	3.112 (11)	152

Symmetry codes: (ii) 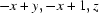; (iii) 
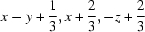
.

**Fig. 1.**
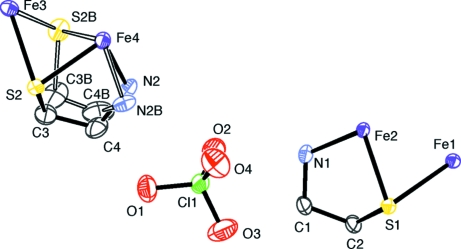
The asymmetric unit of (I), with atom labels and 50% probability displacement ellipsoids for non-H atoms. Hydrogen atoms are omitted for clarity.

**Fig. 2.**
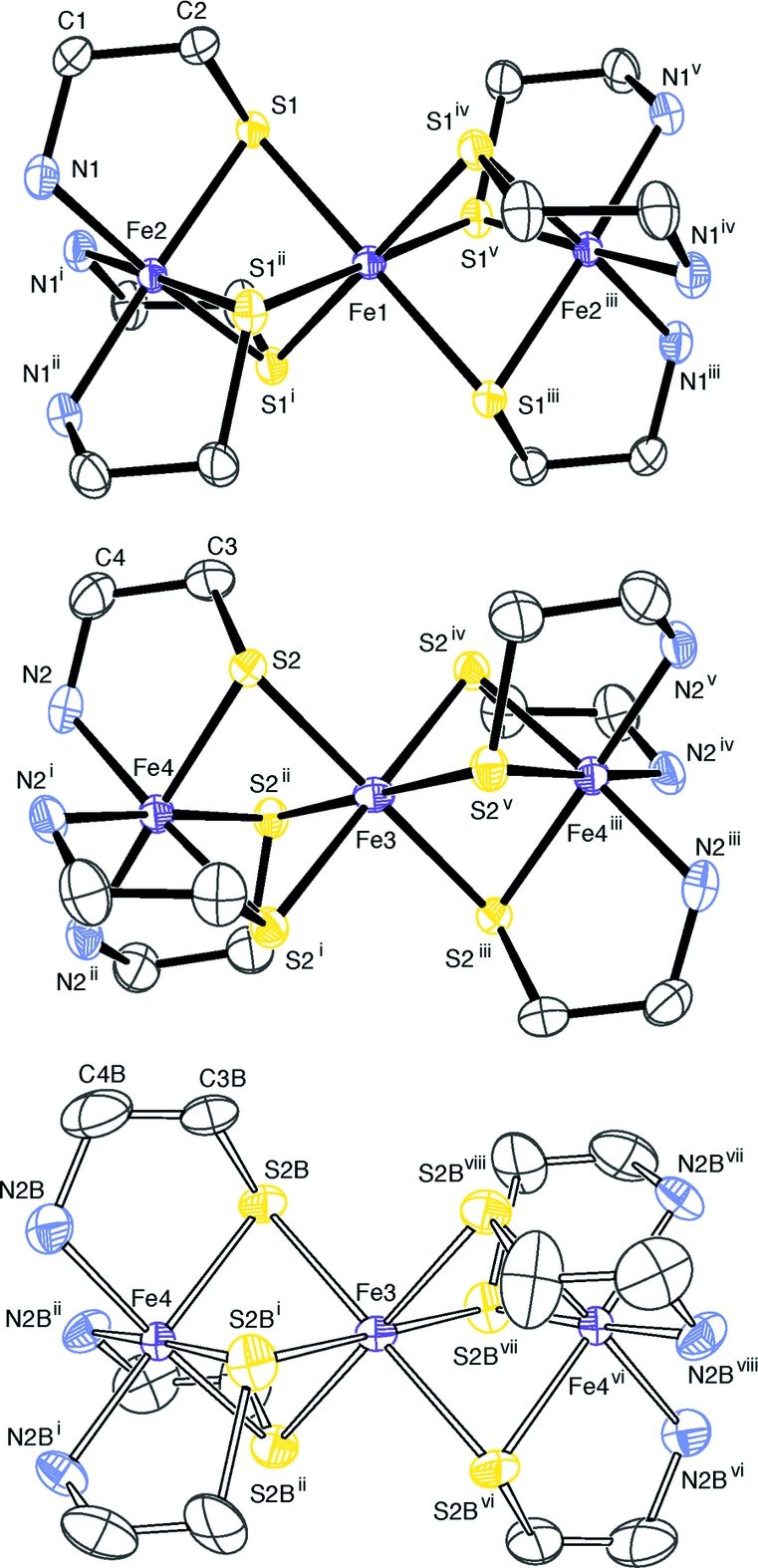
The cation units of (I). The molecule with open bonds is the minor component of the disordered unit. Symmetry codes: (i) -y + 1, x-y + 1, z; (ii) -x + y, -x + 1, z; (iii) -x + 2/3, -y + 4/3, -z + 1/3; (iv) y - 1/3, -x + y+1/3, -z + 1/3; (v) x-y + 2/3, x + 1/3, -z + 1/3; (vi) -x + 2/3, -y + 4/3, -z + 4/3; (vii) y - 1/3, -x + y+1/3, -z + 4/3; (viii) x-y + 2/3, x + 1/3, -z + 4/3.

## supplementary crystallographic information

### Comment

Due to the high Lewis basicity of thiolate groups, a great number of thiolate-bridged complexes have been synthesized up to now. 2-Aminoethanethiolate (aet) is the simplest N,*S*-chelating ligand that has been used for the formation of S-bridged polynuclear structures. For example, it has been shown that the aet ligand reacts with the octahedral metal ions to give linear-type S-bridged trinuclear structures, such as Co^III^_3_ (Busch and Jicha, 1962; Heeg *et al.*, 1985; Marsh *et al.*, 1986), Rh^III^_3_ (Mahboob *et al.*, 2004), and Ru^III^_3_ (Matsuura *et al.*, 2006). In this paper, we report on the crystal structure of the title compound (I), which was obtained by the reaction of aet and Fe(ClO_4_)_3_. The asymmetric unit of the compound (I) contains two complex cations having a threefold rotation-inversion axis and one perchlorate anion. One of the complex cations is disordered over two positions with occupancies of 0.75 and 0.25. In the complex cation of (I), two *fac*(*S*)-[Fe(aet)_3_] units coordinate to a central Fe atom through thiolato bridges to form a linear-type trinuclear structure. Each terminal Fe atom is in an N_3_S_3_ octahedral environment, whereas the central Fe atom is in an S_6_ octahedral environment. Considering the charge balance, it is assumed that all Fe atoms have a +III oxidation state.

### Experimental

To a solution containing 2-aminoethanethiol hydrochloride (0.11 g, 1 mmol) in 20 ml of methanol/CH_2_Cl_2_ (1:1) was added a solution of Et_3_N (0.10 g, 1 mmol) in 10 ml of methanol and a solution of Fe(ClO_4_)_3_.6H_2_O (0.09 g, 0.2 mmol) in 2 ml of methanol. The resulting dark brown solution was stood at room temperature overnight to give black crystals, which was filtered and washed with methanol. Yield: 31 mg (50% based on Fe). Anal. Calcd for [Fe_3_(aet)_6_](ClO_4_)_3_ = C_12_H_36_Cl_3_Fe_3_N_6_O_12_S_6_: C, 15.62; H, 3.93; N, 9.11%. Found: C, 15.82; H, 3.88; N, 9.00%.

### Refinement

H atoms bonded to C and N atoms were placed at calculated positions [C—H = 0.99 and N—H = 0.92 Å] and refined as riding with *U*_iso_(H) = 1.2*U*_eq_ (C,*N*). One cationic part was disordered over two positions (S2, N2, C3, C4 and S2B, N2B, C3B, C4B) and refined with site occupancies of 0.75 and 0.25. The C3B atom in a minor component was restrained based on ISOR.

### Crystal data

**Table:**

[Fe_3_(C_2_H_6_NS)_6_](ClO_4_)_3_	*Z* = 6
*M**_r_* = 922.73	*F*_000_ = 2826
Trigonal, *R*3	*D*_x_ = 1.984 Mg m^−^^3^
Hall symbol: -R 3	Mo *K*α radiation λ = 0.71075 Å
*a* = 14.2852 (6) Å	Cell parameters from 12827 reflections
*b* = 14.2852 Å	θ = 3.4–27.4º
*c* = 26.2187 (8) Å	µ = 2.12 mm^−^^1^
α = 90º	*T* = 200 (2) K
β = 90º	Prism, black
γ = 120º	0.20 × 0.20 × 0.10 mm
*V* = 4633.6 (2) Å^3^	

### Data collection

**Table:**

Rigaku R-AXIS RAPID diffractometer	2365 independent reflections
Radiation source: fine-focus sealed tube	2144 reflections with *I* > 2σ(*I*)
Monochromator: graphite	*R*_int_ = 0.053
Detector resolution: 10.00 pixels mm^-1^	θ_max_ = 27.4º
*T* = 200(2) K	θ_min_ = 3.4º
ω scans	*h* = −18→16
Absorption correction: multi-scan(ABSCOR; Higashi, 1995)	*k* = −18→17
*T*_min_ = 0.677, *T*_max_ = 0.816	*l* = −33→33
15327 measured reflections	

### Refinement

**Table:**

Refinement on *F*^2^	Secondary atom site location: difference Fourier map
Least-squares matrix: full	Hydrogen site location: inferred from neighbouring sites
*R*[*F*^2^ > 2σ(*F*^2^)] = 0.032	H-atom parameters constrained
*wR*(*F*^2^) = 0.100	*w* = 1/[σ^2^(*F*_o_^2^) + (0.0456*P*)^2^ + 3.0528*P*] where *P* = (*F*_o_^2^ + 2*F*_c_^2^)/3
*S* = 1.39	(Δ/σ)_max_ = 0.001
2365 reflections	Δρ_max_ = 1.09 e Å^−^^3^
165 parameters	Δρ_min_ = −0.31 e Å^−^^3^
6 restraints	Extinction correction: none
Primary atom site location: structure-invariant direct methods	

### Special details

**Table:**

Geometry. All e.s.d.'s (except the e.s.d. in the dihedral angle between two l.s. planes) are estimated using the full covariance matrix. The cell e.s.d.'s are taken into account individually in the estimation of e.s.d.'s in distances, angles and torsion angles; correlations between e.s.d.'s in cell parameters are only used when they are defined by crystal symmetry. An approximate (isotropic) treatment of cell e.s.d.'s is used for estimating e.s.d.'s involving l.s. planes.
Refinement. Refinement of *F*^2^ against ALL reflections. The weighted *R*-factor *wR* and goodness of fit *S* are based on *F*^2^, conventional *R*-factors *R* are based on *F*, with *F* set to zero for negative *F*^2^. The threshold expression of *F*^2^ > σ(*F*^2^) is used only for calculating *R*-factors(gt) *etc*. and is not relevant to the choice of reflections for refinement. *R*-factors based on *F*^2^ are statistically about twice as large as those based on *F*, and *R*- factors based on ALL data will be even larger. The 2-aminoethanethiolate ligand of one unit containing Fe3 and Fe4 atoms is disordered over two positions with the occupancies of 0.75 and 0.25. The C3B atom in the minor component is restrained based on ISOR.

### Fractional atomic coordinates and isotropic or equivalent isotropic displacement parameters (Å^2^)

**Table:**

	*x*	*y*	*z*	*U*_iso_*/*U*_eq_	Occ. (<1)
Fe1	0.3333	0.6667	0.1667	0.01712 (18)	
Fe2	0.3333	0.6667	0.269870 (17)	0.01673 (15)	
S1	0.21510 (4)	0.53292 (4)	0.219319 (19)	0.02149 (15)	
N1	0.20358 (15)	0.64420 (15)	0.31253 (7)	0.0255 (4)	
H1	0.2151	0.6336	0.3460	0.031*	
H2	0.1986	0.7059	0.3110	0.031*	
C1	0.09906 (18)	0.5506 (2)	0.29524 (9)	0.0308 (5)	
H3	0.0382	0.5595	0.3071	0.037*	
H4	0.0903	0.4828	0.3100	0.037*	
C2	0.09795 (17)	0.5441 (2)	0.23732 (9)	0.0302 (5)	
H5	0.1026	0.6098	0.2222	0.036*	
H6	0.0306	0.4802	0.2253	0.036*	
Fe3	0.3333	0.6667	0.6667	0.02171 (19)	
Fe4	0.3333	0.6667	0.563330 (19)	0.02155 (16)	
S2	0.20595 (6)	0.53902 (6)	0.61393 (3)	0.02341 (18)	0.75
N2	0.2103 (3)	0.6563 (3)	0.52058 (14)	0.0300 (8)	0.75
H7	0.2163	0.7234	0.5193	0.036*	0.75
H8	0.2176	0.6377	0.4878	0.036*	0.75
C3	0.0975 (3)	0.5650 (4)	0.59679 (17)	0.0394 (9)	0.75
H9	0.0267	0.5041	0.6076	0.047*	0.75
H10	0.1083	0.6319	0.6135	0.047*	0.75
C4	0.1011 (4)	0.5773 (4)	0.5393 (2)	0.0425 (11)	0.75
H11	0.0776	0.5062	0.5233	0.051*	0.75
H12	0.0496	0.6011	0.5289	0.051*	0.75
S2B	0.2622 (3)	0.7412 (2)	0.61334 (10)	0.0335 (5)	0.25
N2B	0.1951 (9)	0.6067 (9)	0.5199 (5)	0.035 (3)	0.25
H13	0.2127	0.6372	0.4879	0.041*	0.25
H14	0.1655	0.5332	0.5163	0.041*	0.25
C3B	0.1180 (11)	0.6301 (17)	0.5950 (6)	0.065 (4)	0.25
H15	0.1036	0.5589	0.6078	0.078*	0.25
H16	0.0638	0.6462	0.6098	0.078*	0.25
C4B	0.1130 (14)	0.6295 (15)	0.5432 (8)	0.060 (5)	0.25
H17	0.1257	0.7006	0.5311	0.072*	0.25
H18	0.0399	0.5737	0.5321	0.072*	0.25
Cl1	0.17461 (5)	0.41937 (4)	0.42062 (2)	0.03377 (17)	
O1	0.12758 (17)	0.38390 (16)	0.47031 (7)	0.0460 (5)	
O2	0.20389 (18)	0.53133 (14)	0.41480 (8)	0.0492 (5)	
O3	0.0981 (2)	0.35574 (17)	0.38241 (8)	0.0604 (6)	
O4	0.26978 (18)	0.41114 (19)	0.41632 (9)	0.0583 (6)	

### Atomic displacement parameters (Å^2^)

**Table:**

	*U*^11^	*U*^22^	*U*^33^	*U*^12^	*U*^13^	*U*^23^
Fe1	0.0171 (2)	0.0171 (2)	0.0173 (4)	0.00853 (12)	0.000	0.000
Fe2	0.01850 (19)	0.01850 (19)	0.0132 (3)	0.00925 (9)	0.000	0.000
S1	0.0233 (3)	0.0207 (3)	0.0166 (3)	0.0081 (2)	−0.00003 (18)	0.00068 (17)
N1	0.0293 (10)	0.0302 (10)	0.0174 (9)	0.0151 (8)	0.0022 (7)	0.0004 (7)
C1	0.0224 (11)	0.0387 (13)	0.0235 (12)	0.0094 (10)	0.0051 (8)	0.0034 (9)
C2	0.0180 (10)	0.0410 (13)	0.0235 (11)	0.0088 (10)	0.0020 (8)	0.0009 (9)
Fe3	0.0190 (2)	0.0190 (2)	0.0272 (4)	0.00950 (12)	0.000	0.000
Fe4	0.0229 (2)	0.0229 (2)	0.0189 (3)	0.01145 (10)	0.000	0.000
S2	0.0220 (4)	0.0236 (4)	0.0205 (4)	0.0082 (3)	−0.0005 (3)	−0.0008 (3)
N2	0.0387 (19)	0.033 (2)	0.0200 (15)	0.0197 (19)	−0.0061 (12)	−0.0045 (16)
C3	0.0233 (18)	0.052 (2)	0.040 (2)	0.0165 (19)	−0.0008 (14)	0.001 (2)
C4	0.031 (2)	0.060 (3)	0.036 (2)	0.023 (3)	−0.0093 (15)	−0.007 (2)
S2B	0.0387 (15)	0.0385 (14)	0.0369 (14)	0.0294 (14)	−0.0028 (11)	0.0001 (11)
N2B	0.034 (5)	0.030 (6)	0.037 (5)	0.014 (6)	−0.011 (4)	−0.014 (5)
C3B	0.026 (6)	0.124 (13)	0.053 (8)	0.043 (9)	−0.010 (5)	−0.014 (10)
C4B	0.033 (7)	0.066 (12)	0.082 (12)	0.026 (9)	−0.018 (7)	−0.014 (11)
Cl1	0.0459 (4)	0.0229 (3)	0.0229 (3)	0.0100 (2)	−0.0001 (2)	0.00124 (19)
O1	0.0606 (13)	0.0428 (11)	0.0322 (10)	0.0239 (10)	0.0109 (9)	0.0091 (8)
O2	0.0767 (15)	0.0243 (9)	0.0382 (12)	0.0188 (9)	−0.0039 (10)	0.0027 (7)
O3	0.0780 (16)	0.0390 (11)	0.0502 (13)	0.0188 (11)	−0.0270 (11)	−0.0113 (9)
O4	0.0501 (12)	0.0571 (13)	0.0655 (15)	0.0252 (11)	0.0103 (10)	0.0076 (11)

### Geometric parameters (Å, °)

**Table:**

Fe1—S1^i^	2.2763 (5)	Fe4—N2^iii^	2.026 (4)
Fe1—S1^ii^	2.2763 (5)	Fe4—N2^iv^	2.026 (4)
Fe1—S1^iii^	2.2764 (5)	Fe4—N2B	2.059 (11)
Fe1—S1	2.2764 (5)	Fe4—N2B^iii^	2.059 (11)
Fe1—S1^iv^	2.2764 (5)	Fe4—N2B^iv^	2.059 (11)
Fe1—S1^v^	2.2764 (5)	Fe4—S2B	2.229 (2)
Fe2—N1	2.0482 (18)	Fe4—S2B^iii^	2.229 (2)
Fe2—N1^iii^	2.0482 (18)	Fe4—S2B^iv^	2.229 (2)
Fe2—N1^iv^	2.0482 (18)	Fe4—S2^iii^	2.2535 (7)
Fe2—S1^iii^	2.2434 (6)	Fe4—S2	2.2535 (8)
Fe2—S1	2.2434 (6)	Fe4—S2^iv^	2.2535 (7)
Fe2—S1^iv^	2.2434 (6)	S2—C3	1.821 (4)
S1—C2	1.821 (2)	N2—C4	1.480 (7)
N1—C1	1.492 (3)	N2—H7	0.9200
N1—H1	0.9200	N2—H8	0.9200
N1—H2	0.9200	C3—C4	1.514 (7)
C1—C2	1.521 (3)	C3—H9	0.9900
C1—H3	0.9900	C3—H10	0.9900
C1—H4	0.9900	C4—H11	0.9900
C2—H5	0.9900	C4—H12	0.9900
C2—H6	0.9900	S2B—C3B	1.929 (16)
Fe3—S2B^vi^	2.281 (2)	N2B—C4B	1.49 (2)
Fe3—S2B	2.281 (2)	N2B—H13	0.9200
Fe3—S2B^iv^	2.281 (2)	N2B—H14	0.9200
Fe3—S2B^iii^	2.281 (2)	C3B—C4B	1.36 (3)
Fe3—S2B^vii^	2.281 (2)	C3B—H15	0.9900
Fe3—S2B^viii^	2.281 (2)	C3B—H16	0.9900
Fe3—S2^viii^	2.2869 (7)	C4B—H17	0.9900
Fe3—S2^iii^	2.2869 (7)	C4B—H18	0.9900
Fe3—S2	2.2869 (7)	Cl1—O3	1.425 (2)
Fe3—S2^iv^	2.2869 (7)	Cl1—O4	1.426 (2)
Fe3—S2^vi^	2.2870 (7)	Cl1—O1	1.4369 (18)
Fe3—S2^vii^	2.2870 (7)	Cl1—O2	1.4447 (18)
Fe4—N2	2.026 (4)		
			
S1^i^—Fe1—S1^ii^	87.042 (18)	N2—Fe4—N2^iii^	92.34 (15)
S1^i^—Fe1—S1^iii^	180.00 (3)	N2—Fe4—N2^iv^	92.34 (15)
S1^ii^—Fe1—S1^iii^	92.959 (18)	N2^iii^—Fe4—N2^iv^	92.34 (15)
S1^i^—Fe1—S1	92.960 (18)	N2B—Fe4—N2B^iii^	92.4 (5)
S1^ii^—Fe1—S1	180.0	N2—Fe4—N2B^iv^	103.6 (3)
S1^iii^—Fe1—S1	87.039 (18)	N2^iii^—Fe4—N2B^iv^	78.4 (3)
S1^i^—Fe1—S1^iv^	92.961 (18)	N2^iv^—Fe4—N2B^iv^	17.7 (3)
S1^ii^—Fe1—S1^iv^	92.961 (18)	N2B—Fe4—N2B^iv^	92.4 (5)
S1^iii^—Fe1—S1^iv^	87.039 (18)	N2B^iii^—Fe4—N2B^iv^	92.4 (5)
S1—Fe1—S1^iv^	87.039 (18)	N2B—Fe4—S2B	87.3 (3)
S1^i^—Fe1—S1^v^	87.041 (18)	N2B^iii^—Fe4—S2B	91.5 (4)
S1^ii^—Fe1—S1^v^	87.040 (18)	N2B^iv^—Fe4—S2B	176.1 (3)
S1^iii^—Fe1—S1^v^	92.959 (18)	N2B—Fe4—S2B^iii^	176.1 (3)
S1—Fe1—S1^v^	92.960 (18)	N2B^iii^—Fe4—S2B^iii^	87.3 (3)
S1^iv^—Fe1—S1^v^	180.0	N2B^iv^—Fe4—S2B^iii^	91.5 (4)
N1—Fe2—N1^iii^	93.02 (7)	S2B—Fe4—S2B^iii^	88.91 (9)
N1—Fe2—N1^iv^	93.02 (7)	N2B—Fe4—S2B^iv^	91.5 (4)
N1^iii^—Fe2—N1^iv^	93.02 (7)	N2B^iii^—Fe4—S2B^iv^	176.1 (3)
N1—Fe2—S1^iii^	91.05 (5)	N2B^iv^—Fe4—S2B^iv^	87.3 (3)
N1^iii^—Fe2—S1^iii^	87.26 (5)	S2B—Fe4—S2B^iv^	88.91 (9)
N1^iv^—Fe2—S1^iii^	175.90 (5)	S2B^iii^—Fe4—S2B^iv^	88.91 (9)
N1—Fe2—S1	87.26 (5)	N2—Fe4—S2^iii^	91.91 (11)
N1^iii^—Fe2—S1	175.90 (5)	N2^iii^—Fe4—S2^iii^	86.94 (11)
N1^iv^—Fe2—S1	91.05 (5)	N2^iv^—Fe4—S2^iii^	175.71 (11)
S1^iii^—Fe2—S1	88.65 (2)	N2—Fe4—S2	86.94 (11)
N1—Fe2—S1^iv^	175.90 (5)	N2^iii^—Fe4—S2	175.71 (11)
N1^iii^—Fe2—S1^iv^	91.05 (5)	N2^iv^—Fe4—S2	91.91 (11)
N1^iv^—Fe2—S1^iv^	87.26 (5)	S2^iii^—Fe4—S2	88.86 (3)
S1^iii^—Fe2—S1^iv^	88.65 (2)	N2—Fe4—S2^iv^	175.71 (11)
S1—Fe2—S1^iv^	88.65 (2)	N2^iii^—Fe4—S2^iv^	91.91 (11)
C2—S1—Fe2	96.07 (8)	N2^iv^—Fe4—S2^iv^	86.94 (11)
C2—S1—Fe1	114.36 (8)	S2^iii^—Fe4—S2^iv^	88.86 (3)
Fe2—S1—Fe1	73.546 (18)	S2—Fe4—S2^iv^	88.86 (3)
C1—N1—Fe2	113.33 (14)	C3—S2—Fe4	96.60 (14)
C1—N1—H1	108.9	C3—S2—Fe3	113.93 (15)
Fe2—N1—H1	108.9	Fe4—S2—Fe3	73.27 (2)
C1—N1—H2	108.9	C4—N2—Fe4	114.7 (3)
Fe2—N1—H2	108.9	C4—N2—H7	108.6
H1—N1—H2	107.7	Fe4—N2—H7	108.6
N1—C1—C2	109.46 (17)	C4—N2—H8	108.6
N1—C1—H3	109.8	Fe4—N2—H8	108.6
C2—C1—H3	109.8	H7—N2—H8	107.6
N1—C1—H4	109.8	C4—C3—S2	106.6 (3)
C2—C1—H4	109.8	C4—C3—H9	110.4
H3—C1—H4	108.2	S2—C3—H9	110.4
C1—C2—S1	106.38 (15)	C4—C3—H10	110.4
C1—C2—H5	110.5	S2—C3—H10	110.4
S1—C2—H5	110.5	H9—C3—H10	108.6
C1—C2—H6	110.5	N2—C4—C3	112.4 (4)
S1—C2—H6	110.5	N2—C4—H11	109.1
H5—C2—H6	108.6	C3—C4—H11	109.1
S2B^vi^—Fe3—S2B	179.998 (1)	N2—C4—H12	109.1
S2B^vi^—Fe3—S2B^iv^	93.64 (9)	C3—C4—H12	109.1
S2B—Fe3—S2B^iv^	86.37 (9)	H11—C4—H12	107.9
S2B^vi^—Fe3—S2B^iii^	93.64 (9)	C3B—S2B—Fe4	90.9 (5)
S2B—Fe3—S2B^iii^	86.37 (9)	C3B—S2B—Fe3	108.1 (6)
S2B^iv^—Fe3—S2B^iii^	86.37 (9)	Fe4—S2B—Fe3	73.83 (7)
S2B^vi^—Fe3—S2B^vii^	86.36 (9)	C4B—N2B—Fe4	112.1 (9)
S2B—Fe3—S2B^vii^	93.63 (9)	C4B—N2B—H13	109.2
S2B^iv^—Fe3—S2B^vii^	180.00 (10)	Fe4—N2B—H13	109.2
S2B^iii^—Fe3—S2B^vii^	93.64 (9)	C4B—N2B—H14	109.2
S2B^vi^—Fe3—S2B^viii^	86.36 (9)	Fe4—N2B—H14	109.2
S2B—Fe3—S2B^viii^	93.63 (9)	H13—N2B—H14	107.9
S2B^iv^—Fe3—S2B^viii^	93.64 (9)	C4B—C3B—S2B	106.6 (13)
S2B^iii^—Fe3—S2B^viii^	179.998 (1)	C4B—C3B—H15	110.4
S2B^vii^—Fe3—S2B^viii^	86.36 (9)	S2B—C3B—H15	110.4
S2^viii^—Fe3—S2^iii^	180.00 (5)	C4B—C3B—H16	110.4
S2^viii^—Fe3—S2	92.77 (3)	S2B—C3B—H16	110.4
S2^iii^—Fe3—S2	87.23 (3)	H15—C3B—H16	108.6
S2^viii^—Fe3—S2^iv^	92.77 (3)	C3B—C4B—N2B	111.5 (14)
S2^iii^—Fe3—S2^iv^	87.23 (3)	C3B—C4B—H17	109.3
S2—Fe3—S2^iv^	87.23 (3)	N2B—C4B—H17	109.3
S2^viii^—Fe3—S2^vi^	87.23 (3)	C3B—C4B—H18	109.3
S2^iii^—Fe3—S2^vi^	92.77 (3)	N2B—C4B—H18	109.3
S2—Fe3—S2^vi^	180.0	H17—C4B—H18	108.0
S2^iv^—Fe3—S2^vi^	92.77 (3)	O3—Cl1—O4	110.49 (15)
S2^viii^—Fe3—S2^vii^	87.23 (3)	O3—Cl1—O1	109.78 (14)
S2^iii^—Fe3—S2^vii^	92.77 (3)	O4—Cl1—O1	109.85 (13)
S2—Fe3—S2^vii^	92.77 (3)	O3—Cl1—O2	109.71 (13)
S2^iv^—Fe3—S2^vii^	179.999 (1)	O4—Cl1—O2	108.82 (14)
S2^vi^—Fe3—S2^vii^	87.23 (3)	O1—Cl1—O2	108.16 (12)
			
N1—Fe2—S1—C2	21.81 (10)	S2^viii^—Fe3—S2—Fe4	−136.322 (12)
N1^iv^—Fe2—S1—C2	114.79 (9)	S2^iii^—Fe3—S2—Fe4	43.680 (12)
S1^iii^—Fe2—S1—C2	−69.30 (8)	S2^iv^—Fe3—S2—Fe4	−43.680 (12)
S1^iv^—Fe2—S1—C2	−157.98 (8)	S2^vii^—Fe3—S2—Fe4	136.320 (12)
N1—Fe2—S1—Fe1	135.45 (5)	N2^iii^—Fe4—N2—C4	−175.7 (3)
N1^iv^—Fe2—S1—Fe1	−131.57 (5)	N2^iv^—Fe4—N2—C4	91.9 (4)
S1^iii^—Fe2—S1—Fe1	44.341 (11)	S2^iii^—Fe4—N2—C4	−88.6 (3)
S1^iv^—Fe2—S1—Fe1	−44.341 (11)	S2—Fe4—N2—C4	0.1 (3)
S1^i^—Fe1—S1—C2	−134.32 (9)	Fe4—S2—C3—C4	44.5 (3)
S1^iii^—Fe1—S1—C2	45.68 (9)	Fe3—S2—C3—C4	119.0 (3)
S1^iv^—Fe1—S1—C2	132.87 (8)	Fe4—N2—C4—C3	30.6 (5)
S1^v^—Fe1—S1—C2	−47.13 (8)	S2—C3—C4—N2	−51.4 (5)
S1^i^—Fe1—S1—Fe2	136.408 (8)	N2B—Fe4—S2B—C3B	27.4 (7)
S1^iii^—Fe1—S1—Fe2	−43.592 (8)	N2B^iii^—Fe4—S2B—C3B	119.7 (7)
S1^iv^—Fe1—S1—Fe2	43.592 (8)	S2B^iii^—Fe4—S2B—C3B	−153.1 (6)
S1^v^—Fe1—S1—Fe2	−136.406 (8)	S2B^iv^—Fe4—S2B—C3B	−64.1 (6)
N1^iii^—Fe2—N1—C1	−179.13 (15)	N2B—Fe4—S2B—Fe3	136.0 (4)
N1^iv^—Fe2—N1—C1	−85.94 (19)	N2B^iii^—Fe4—S2B—Fe3	−131.7 (3)
S1^iii^—Fe2—N1—C1	93.56 (15)	S2B^iii^—Fe4—S2B—Fe3	−44.47 (4)
S1—Fe2—N1—C1	4.96 (15)	S2B^iv^—Fe4—S2B—Fe3	44.47 (4)
Fe2—N1—C1—C2	−37.5 (2)	S2B^iv^—Fe3—S2B—C3B	42.2 (5)
N1—C1—C2—S1	57.4 (2)	S2B^iii^—Fe3—S2B—C3B	128.8 (5)
Fe2—S1—C2—C1	−46.89 (16)	S2B^vii^—Fe3—S2B—C3B	−137.8 (5)
Fe1—S1—C2—C1	−121.55 (14)	S2B^viii^—Fe3—S2B—C3B	−51.2 (5)
N2—Fe4—S2—C3	−23.37 (18)	S2B^iv^—Fe3—S2B—Fe4	−43.29 (4)
N2^iv^—Fe4—S2—C3	−115.61 (18)	S2B^iii^—Fe3—S2B—Fe4	43.29 (4)
S2^iii^—Fe4—S2—C3	68.61 (15)	S2B^vii^—Fe3—S2B—Fe4	136.71 (4)
S2^iv^—Fe4—S2—C3	157.50 (15)	S2B^viii^—Fe3—S2B—Fe4	−136.71 (4)
N2—Fe4—S2—Fe3	−136.42 (10)	N2B^iii^—Fe4—N2B—C4B	−96.9 (14)
N2^iv^—Fe4—S2—Fe3	131.34 (11)	N2B^iv^—Fe4—N2B—C4B	170.6 (12)
S2^iii^—Fe4—S2—Fe3	−44.441 (14)	S2B—Fe4—N2B—C4B	−5.5 (11)
S2^iv^—Fe4—S2—Fe3	44.441 (14)	S2B^iv^—Fe4—N2B—C4B	83.3 (11)
S2^viii^—Fe3—S2—C3	133.80 (16)	Fe4—S2B—C3B—C4B	−54.2 (13)
S2^iii^—Fe3—S2—C3	−46.20 (16)	Fe3—S2B—C3B—C4B	−127.5 (12)
S2^iv^—Fe3—S2—C3	−133.55 (16)	S2B—C3B—C4B—N2B	59.4 (18)
S2^vii^—Fe3—S2—C3	46.45 (16)	Fe4—N2B—C4B—C3B	−32.3 (19)

Symmetry codes: (i) *x*−*y*+2/3, *x*+1/3, −*z*+1/3; (ii) −*x*+2/3, −*y*+4/3, −*z*+1/3; (iii) −*x*+*y*, −*x*+1, *z*; (iv) −*y*+1, *x*−*y*+1, *z*; (v) *y*−1/3, −*x*+*y*+1/3, −*z*+1/3; (vi) −*x*+2/3, −*y*+4/3, −*z*+4/3; (vii) *y*−1/3, −*x*+*y*+1/3, −*z*+4/3; (viii) *x*−*y*+2/3, *x*+1/3, −*z*+4/3.

### Hydrogen-bond geometry (Å, °)

**Table:**

*D*—H···*A*	*D*—H	H···*A*	*D*···*A*	*D*—H···*A*
N1—H1···O2	0.92	2.28	3.130 (3)	154
N1—H2···O3^ix^	0.92	2.40	3.162 (3)	140
N2—H7···O1^iii^	0.92	2.30	3.110 (4)	147
N2—H8···O2	0.92	2.39	3.274 (4)	161
N2B—H13···O2	0.92	2.41	2.984 (12)	121
N2B—H14···O1	0.92	2.27	3.112 (11)	152

Symmetry codes: (ix) *x*−*y*+1/3, *x*+2/3, −*z*+2/3; (iii) −*x*+*y*, −*x*+1, *z*.
